# Differentiation of vestibular schwannomas from meningiomas of the internal auditory canal using perilymphatic signal evaluation on T2-weighted gradient-echo fast imaging employing steady state acquisition at 3T

**DOI:** 10.1186/s41747-017-0012-7

**Published:** 2017-06-29

**Authors:** A. Venkatasamy, D. Le Foll, A. Karol, B. Lhermitte, A. Charpiot, C. Debry, F. Proust, N. Meyer, F. Veillon

**Affiliations:** 1 Imagerie 1, Radiologie, Hôpitaux Universitaires de Strasbourg, Strasbourg, France; 2 Service d’Anatomie Pathologique, Hôpitaux Universitaires de Strasbourg, Strasbourg, France; 3 Service d’ORL, Hôpitaux Universitaires de Strasbourg, Strasbourg, France; 4 Service de Neurochirurgie, Hôpitaux Universitaires de Strasbourg, Strasbourg, France; 5 Département de Statistiques, Hôpitaux Universitaires de Strasbourg, Strasbourg, France

**Keywords:** Schwannoma, Meningioma, Magnetic resonance imaging (MRI), Perilymph, Internal auditory canal

## Abstract

**Background:**

Our aim was to confirm the usefulness of the perilymphatic signal changes on T2-weighted (T2W) gradient-echo sequence to differentiate vestibular schwannomas from internal auditory canal (IAC) meningiomas, through a compartmental analysis of inner ear fluids signal intensity.

**Methods:**

A total of 203 patients with all criteria for typical vestibular schwannoma on T1-weighted contrast-enhanced sequences were retrospectively enrolled (190 schwannomas and 13 meningiomas). All patients underwent a T2W gradient-echo steady state free precession (SSFP) acquisition at 3T. Two radiologists analysed the signal intensity of the perilymph (cistern and cochlea) and endolymph (saccule and utricle) using a region of interest-based method for obtaining ratios between the analysed structures and the cerebrospinal fluid (CSF).

**Results:**

Obstructive vestibular schwannomas showed a markedly decreased perilymphatic signal in both cistern and cochlea; the cistern/CSF ratio (Ci/CSF) was 0.62. The decrease was more moderate in IAC meningiomas (Ci/CSF = 0.81). For Ci/CSF > 0.70, the tumour was more likely a meningioma, with a 92% sensitivity and 83% specificity. No endolymphatic signal changes were observed.

**Conclusion:**

The pronounced decrease in perilymphatic signal on a T2W SSFP sequence in obstructive vestibular schwannoma provides a new tool to differentiate schwannomas from IAC meningiomas, which may be useful to overcome the insufficiencies of morphological analysis.

## Key points

- The differentiation between schwannomas and meningiomas is important for the patient’s care.
- Perilymphatic signal decrease in vestibular schwannomas on T2-weighte images is a reliable new diagnostic tool.
- For a Ci/CSF ratio higher than 0.70, the tumour was more likely a meningioma.
- No significant changes of the intensity of the endolymphatic signal were observed.
- For IAC tumours, the MRI reading should include analysis of inner ear fluids.

## Background

The two most frequent cerebello-pontine angle tumours are vestibular schwannomas (80%) and meningiomas (10–15%). Only 10–22% of cerebellopontine angle meningiomas extend into the internal auditory canal (IAC) or arise from its dural lining [1–5]. Differentiating these tumours is essential for their treatment, especially in the choice of the surgical approach: an enlarged translabyrinthine approach is favoured for vestibular schwannomas, while a retrosigmoid approach is recommended for cerebellopontine angle meningiomas [3, 4, 6–9].

MRI with an axial T1-weighted (T1W) contrast-enhanced sequence after intravenous injection of a gadolinium-based contrast agent is the common imaging protocol for cerebello-pontine angle tumours. A majority of tumours present typical magnetic resonance imaging (MRI) features, but some of them may have similar imaging findings, which render their diagnosis uncertain [10–12]. Unlike T1W contrast-enhanced imaging, steady state free precession (SSFP) sequences — such as the fast imaging employing steady-state acquisition with cycle phase (FIESTA-C) sequence — allow an optimal study of the signal of the internal ear liquids and is also sensitive to the composition of inner ear fluids. Vestibular schwannomas and IAC meningiomas are known to have the propensity to increase protein concentration in the cerebrospinal fluid (CSF) and the perilymph, with different protein concentration according to the tumour type [13–17]. Three research teams have studied the signal changes in either the anterior or the posterior labyrinth of patients with vestibular schwannomas on fluid attenuated inversion recovery (FLAIR) or SSFP sequences [18–21], but the endolymphatic signal in cerebello-pontine angle tumours and a compartmental analysis of both endolymph and perilymph have not yet been studied.

The aim of this study was to evaluate the signal changes of the endolymph and perilymph in vestibular schwannomas and IAC meningiomas, compared with a group of healthy volunteers by means of a compartmental analysis of the signal intensity of the saccule, utricle, vestibular cistern and cochlea on a T2-weighted (T2W) gradient-echo FIESTA-C sequence.

## Methods

The Ethics Committee of our institution approved the study (authorization number 5012). The patients gave their consent to participate to the study.

### Study population

Patients who presented at our institution with either a possible diagnosis of vestibular schwannoma or IAC meningioma from 2008 to 2016 were retrospectively enrolled. A total of 262 patients were eligible, but we only selected those meeting all criteria for typical vestibular schwannoma or typical IAC meningioma on a T1W contrast-enhanced sequence acquired on a 3T MRI (see Table 1) [10–12, 18]. Fifty-nine patients were excluded. The exclusion criteria were: insufficient imaging data; previously treated tumours; atypical tumours for which the diagnosis was unclear; intralabyrinthine schwannomas or intralabyrinthine tumour extension (tumours spreading to the labyrinth – endolymph and perilymph – on T1W contrast-enhanced images); and concomitant infections or inflammatory inner/middle ear pathologies. Of the 203 patients included, 23 (11%) had a surgical confirmation of the diagnosis of schwannoma (n = 18) or IAC meningioma (n = 5) given on T1W contrast-enhanced images. In all cases, the absence of an intralabyrinthine extension of the tumour was also surgically confirmed.

**Table 1:** Axial T1W contrast-enhanced-based seven-item checklist for the diagnosis of typical vestibular schwannoma versus IAC meningioma

	IAC centric^a^	Anterior extension^b^ > 1 cm	IAC dilation^c^	Tumour axis perpendicular to petrous bone^d^	Tumour axis parallel to petrous bone^e^	Acute tumour-bone angle^f^	Dural tail^g^
VS	+	-	+	+	-	+	-
IAC M	-	+	-	-	+	-	+

*VS* vestibular schwannoma, *IAC M* internal auditory canal meningioma

^a^Centric location of the tumour to the IAC

^b^Extension of the tumour further than 1 cm from the anterior wall of the IAC

^c^Dilation of the IAC by the tumour

^d^The tumour’s axis is perpendicular to the petrous bone

^e^The tumour’s axis is parallel to the petrous bone

^f^The angle between the tumour and the posterior wall of the petrous bone is acute

^g^Thickening and enhancement of the meninges adjacent to the tumour

As mentioned above, 203 patients (190 with typical vestibular schwannomas and 13 with typical IAC meningiomas) were included. The 190 vestibular schwannomas were subdivided into three groups (groups 1, 2, and 3) prior to the analysis, according to the degree of obstruction of the IAC. Group 1 comprised 33 non-obstructive vestibular schwannomas: these schwannomas presented with more than 1 mm of CSF around the tumour in the IAC. Group 2 was composed of 31 CSF-border vestibular schwannomas: they presented with a small border of CSF (≥1 mm) between the tumour and the IAC walls. The 126 obstructive vestibular schwannomas, which were included in group 3, completely obstructed the IAC without visible CSF around the tumour. Thirteen obstructive IAC meningiomas, with unquestionable imaging features, either extending into the IAC from the cerebello-pontine angle (n = 10) or originating primarily in the IAC (n = 3), were included in group 4.

The patients were compared with 64 healthy volunteers who served as a control group (33 women, 31 men; mean age = 32.3 years, age range = 22–57 years). Healthy volunteers did not present any past or present otological symptoms. The healthy volunteers control group used in this study was initially designed for another study (aimed at comparing histological sections with MRI images). The Ethics Committee agreed on the use of the FIESTA-C sequence (unenhanced scan lasting 7 min 49 s) for the volunteers of the control group. Thus, the control group was prospectively created and used for both the original and the current study (in the latter case after a new authorization by the Ethics Committee).

### MRI protocol

All participants (203 patients and the control group) underwent an axial high-resolution T2W three-dimensional (3D) gradient-echo FIESTA-C sequence at 3T (Signa HDxt, General Electric, Strasbourg, France), using an eight-channel head coil. This sequence is a sort of modified steady state free precession sequence, which does not require any contrast injection. The study box was placed parallel to the orbital roof, extended from the orbital roof, downwards on 2.3 cm. The acquisition parameters were as follows: echo time (TE) = 0.8–1.2 ms, repetition time (TR) = 7 ms, field of view (FOV) = 22× 19.80 cm, frequency × phase = 484 × 484, flip angle = 60°, number of excitations (NEX) = 1, bandwidth = 83.3 kHz, partition thickness = 0.3 × 0.3 × 0.3 mm (isotropic voxel). The acquisition time was 7 min 49 s.

The 203 patients underwent an additional axial contrast-enhanced two-dimensional spin-echo T1W sequence, centred on the IAC, 10 min after intravenous injection of 0.1 mmol/kg (0.2 ml/kg) of gadoterate meglumine (Dotarem®, Guerbet, Roissy, France) because the imaging diagnosis was based on the morphological features of the tumour on this sequence (see Table 1). The acquisition parameters were as follows: TE = 14 ms, TR = 580 ms, FOV = 20 × 20 cm, frequency × phase = 320 × 320, FA = 90°, NEX = 2, bandwidth = 1.23 kHz, slice thickness = 1 mm. The acquisition time was 6 min 15 s.

### Visual analysis

Coronal and sagittal reconstructions were obtained from the original axial dataset of the 3D T2W sequence. All measurements were carried out using the open-source OsiriX software (available at: http://www.osirix-viewer.com/). A 5th-year radiology resident and a senior radiologist with 35 years of experience in head and neck imaging read the high-resolution T2W gradient-echo sequence, blinded to the results of the T1W contrast-enhanced sequence. They visually analysed the signal intensity of the perilymph (vestibular cistern and cochlea) and endolymph (saccule and utricle) on the side of the tumour in comparison with those on the normal side, as well as CSF signal in the cerebello-pontine angle on the side of the tumour. The signal intensity was rated on a three-level scale from 0 to 2 (0 = if judged similar to the contralateral normal side and to the CSF of the adjacent cerebello-pontine angle; 1 = if intermediate, i.e. neither as bright as the contralateral ear’s signal and the surrounding CSF nor as hypointense as the tumour [all 203 tumors were hypointense in the study]; 2 = if judged clearly abnormal, i.e. as hypointense as the tumour signal).

### Quantitative analysis

**Fig. 1:**
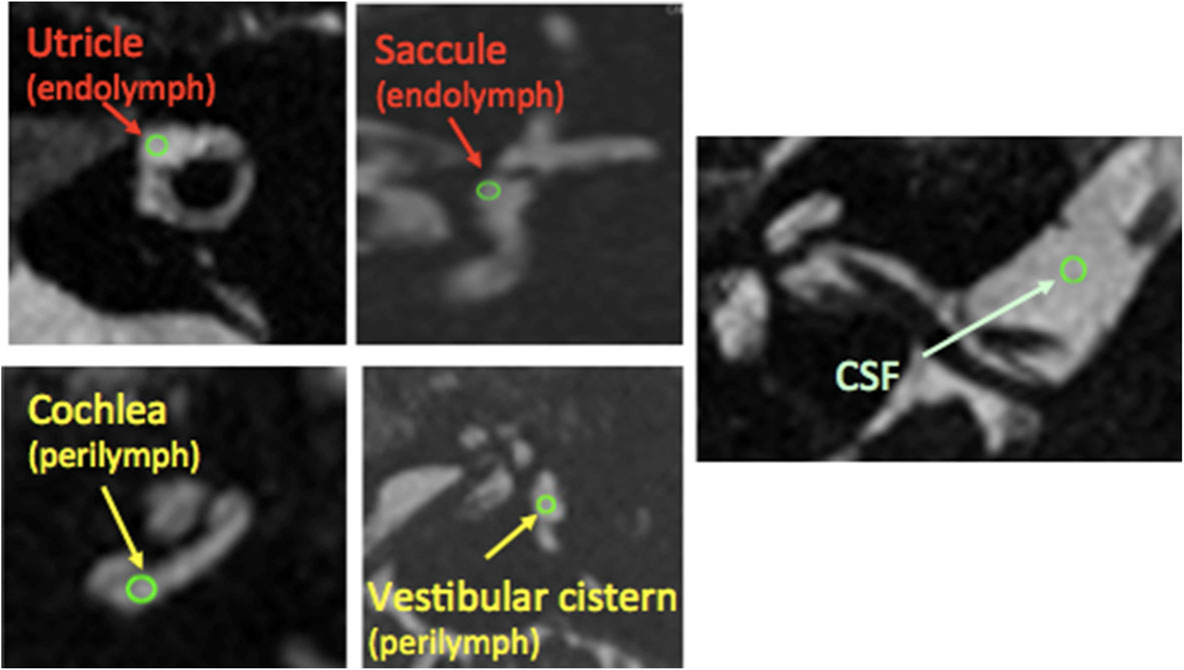
Visual example of the placement of ROIs for the quantitative analysis of the inner ear fluids’ signal. For each labyrinth, a 1-mm^2^ ROI was placed on an axial section in the utricle (endolymph, *red arrow*). A 0.5-mm^2^ ROI was positioned on a coronal section in the saccule (endolymph, *red arrow*). A 1-mm^2^ ROI was placed in both the vestibular cistern and the tympanic portion of the basal turn of the cochlea (perilymph, *yellow arrow*). An additional 1.5-mm^2^ ROI was placed in the CSF of the homolateral CPA (*green arrow*)

For the quantitative analysis, the signal intensity of the endolymph and perilymph was measured using elliptical regions of interests (ROIs) based on a compartmental reading of the FIESTA-C sequence. For each labyrinth, a 1-mm^2^ ROI was placed on an axial section in the utricle, the vestibular cistern and the tympanic portion of the basal turn of the cochlea. An additional 1.5-mm^2^ ROI was placed in the CSF of the ipsilateral cerebello-pontine angle. A 0.5-mm^2^ ROI was positioned on a coronal section in the saccule. The signal intensity ratios of the saccule, utricle, vestibular cistern and cochlea to that of the signal intensity of the CSF (S/CSF, U/CSF, Ci/CSF, Co/CSF, respectively) was calculated in order to avoid bias due to flow artifacts or patient-related artifacts. An example of the placement of the ROIs in the different compartments of the inner ear is illustrated in Fig. 1.

### Otological assessments

Clinical data were available for 185/203 patients (91%). A survey was filled either by the referring otorhinolaryngologist, the neurosurgeon or the general practitioner of the patient; if necessary, data were gathered directly from the clinical records. The collected data included: type of hearing loss (conductive, sensorineural, mixed); progressive or acute nature of the deafness; presence or absence of tinnitus; dizziness or balance disorders. If available, the hearing thresholds of 250–500–1000–2000–4000–8000 Hz frequencies of the tonal audiogram were used to calculate the mean degree of hearing loss.

### Statistical analysis

Continuous variables were expressed as means with their standard deviation (SD). Categorical variables were expressed in terms of numbers and percentages. The inter-observer agreement for the visual analysis was calculated using Cohen’s κ. Inferential statistical analyses were conducted using Bayesian methods [22, 23]. The signal data were repeated since each examination was read twice by each reader. The analysis used linear mixed models with a fixed group effect, fixed reader effect and a random subject effect. Results for groups are expressed as averaged over the two readers. Mean differences were estimated using normal lowly informative prior distribution (N [μ = 0,σ = 10]) [24].

The results are presented as a mean with SD or as a two-groups mean difference and its 95% credibility interval, with the probability that the mean difference is positive, i.e. Pr(diff > 0). Larger values of this probability (near 1) indicate a higher likelihood of the difference being positive. A probability approaching 0.5 must be interpreted as the absence of difference. It should be noted that the Bayesian analysis does not use a (frequentist) *p* value and this probability must not be confused with a *p* value. To estimate the discrimination performances of Ci/CSF ratio and Co/CSF ratio, receiver operating characteristic (ROC) curves were fitted with the pROC R package (pROC 1.8 for R, available at http://web.expasy.org/pROC/)) [25]. Sensitivity, specificity, positive and negative predictive values were computed based on the optimal threshold value (Youden point) and the area under the curve (AUC) was provided for each ROC curve. The Bayesian analyses were run with JAGS software (version 4.2.0.) and R software (version 3.2.0, available at https://www.r-project.org/) [26, 27]. After a burn-in of 5000 updates, 100,000 iterations were performed and convergence was checked using trace plots of the sample values for each iteration.

## Results

The clinical features of all patients and healthy volunteers are given in Table 2.

**Table 2:** Clinical and otological features of patients from the five study groups

	Control group (group 1)	Non-obstructive vestibular schwannoma (group 2)	CSF-border vestibular schwannoma (group 3)	Obstructive vestibular schwannoma (group 4)	IAC meningioma (group 5)
Patients (n)	60	33	31	126	13
Mean tumour volume (cm^3^) [range]	NA	0.03 [0.05–0.247]	0.31 [0.03–1.023]	1.49 [0.03–20]	3.74 [0.16–20.14]
				T1: 0.219	
				T2: 0.54	
				T3: 2.83	
				T4: 11.43	
M : F	31 : 33	14 : 19	13 : 18	68 : 58	2 : 11
Mean age (years) [range]	32.3 [22–57]	63 [41–80]	61 [26–86]	58 [24–92]	66 [39–85]
Hearing loss	0	89% (n = 24)	96% (n = 25)	89% (n = 111)	67% (n = 8)
Perception	0	88% (n = 21)	100% (n = 25)	89% (n = 99)	100% (n = 8)
Mixed	0	12% (n = 3)	0	11% (12)	0%
Progressive	0	88% (n = 21)	92% (n = 23)	78% (n = 87)	100% (n = 8)
Sudden	0	12% (n = 3)	8% (n = 2)	22% (n = 24)	0%
Mean hearing loss (dB)	0	36	45	48	26
Tinnitus	0	40% (n = 11)	69% (n = 18)	50% (n = 61)	50% (n = 6)
Vertigo	0	44% (n = 12)	31% (n = 8)	34% (n = 42)	60% (n = 8)
Dizziness	0	19% (n = 5)	25% (n = 6)	37% (n = 45)	17% (n = 2)

*NA* not applicable, *T1-T2-T3-T4* Morphological classification of the schwannomas of the eighth nerve according Portmann and Bebear [35]

### Visual analysis

**Fig. 2:**
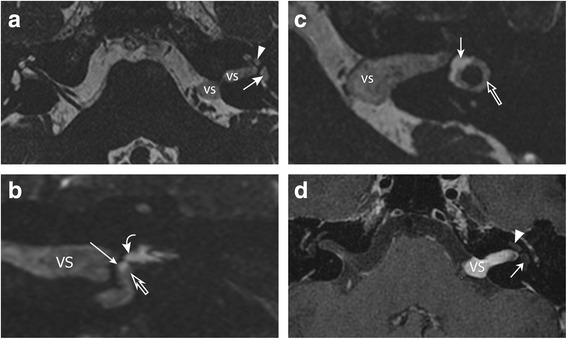
**a** Axial T2W gradient-echo FIESTA-C in an obstructive VS of the left internal auditory canal. There is an important decrease of the perilymphatic signal, which was rated 2, compared with surrounding CSF and the contralateral side, in both the cistern (*arrow*) and the cochlea (*arrow head*). **b** Same patient. Coronal T2W gradient-echo FIESTA-C in an obstructive vestibular schwannoma. This coronal sequence also shows the marked drop of the perilymphatic signal in the cistern (*empty arrow*). The endolymphatic signal in both the saccule (*arrow*) and the utricle (*curved arrow*) appears normal. **c** Same patient. Axial T2W gradient-echo FIESTA-C showing the normal signal of the utricle (*arrow*). Note that the signal of perilymph the lateral semi-circular canal (*empty arrow*) is also decreased, even though we chose not to analyse this perilymphatic compartment individually. **d** Same patient. Axial T1W image after intravenous gadolinium injection, showing the vestibular schwannoma in the IAC and confirming the absence of intralabyrinthine extension of the tumour

On the visual assessment, the lowest signal intensity of the perilymph in both cochlea and cistern were detected in obstructive vestibular schwanommas compared with the contralateral side and surrounding CSF. In obstructive vestibular schwannomas, the perilymph on the pathological side always appeared as hypointense as the tumour on the FIESTA-C sequence and was thus rated 2 in all 126 patients (100%), with an excellent agreement between observers (κ = 1.0) (Fig. 2).

**Fig. 3:**
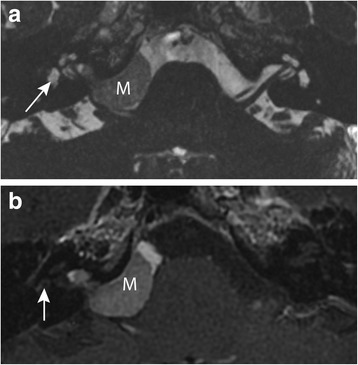
**a** Axial T2W gradient-echo FIESTA-C in an obstructive meningioma of the right internal auditory canal, presenting with a very moderate drop of the perilymphatic signal in the cistern (*arrow*) and the cochlea, rated 1, compared with the normal contralateral side and surrounding CSF. **b** Same patient. Axial section T1W sequence after intravenous gadolinium injection showing the typical meningioma in the IAC

On the contrary, in the IAC meningiomas group, the signal intensity of the perilymph in both cochlea and cistern was either normal (rated 0) or only slightly lower compared with the normal contralateral side (rated 1), with a good agreement between observers (κ = 0.698) (Fig. 3). In fact, no IAC meningiomas was rated 2 by each of the two observers, as their perilymphatic signal was never as low as the signal of the tumour, as it was observed in 100% of our vestibular schwannomas cases.

The decrease of the perilymphatic signal in the three vestibular schwannomas groups (groups 1, 2, and 3) appeared to be correlated to the degree of obstruction of the IAC by the tumour. The obstructive vestibular schwannomas (group 3) presented with the more pronounced decrease of perilymphatic signal on the tumour side (rated 2 in 100% of the patients), while the less obstructive CSF-border vestibular schwannomas (group 2) presented with a perilymphatic signal ranging from 0 (12 patients) to 1 (11 patients) or 2 (eight patients) with an excellent inter-observer agreement (κ = 1.0). The non-obstructive vestibular schwannomas (group 2) did not present any signal changes of the perilymphatic signal (kappa = 1). In the control group, the perilymphatic signal was completely normal on both sides. There were no signal changes of the endolymph in all the studied groups, regardless of the type of tumour and degree of obstruction.

### Quantitative analysis

Obstructive vestibular schwannomas presented with a marked signal decrease of the perilymph of the vestibular cistern with a Ci/CSF ratio of 0.62 ± 0.07 (mean ± SD) to be compared with 0.99 ± 0.08 in the control group. The probability that the mean difference was positive (Pr(diff > 0) = 1, with a credibility interval [−0.38, −0.33]). Obstructive vestibular schwannomas also presented with a marked signal decrease of the perilymph of the cochlea with a mean signal intensity Co/CSF ratio of 0.54 ± 0.07 to be compared with 0.92 ± 0.09 in the control group (Pr(diff > 0) = 1, [−0.06, 0.02]).

**Fig. 4:**
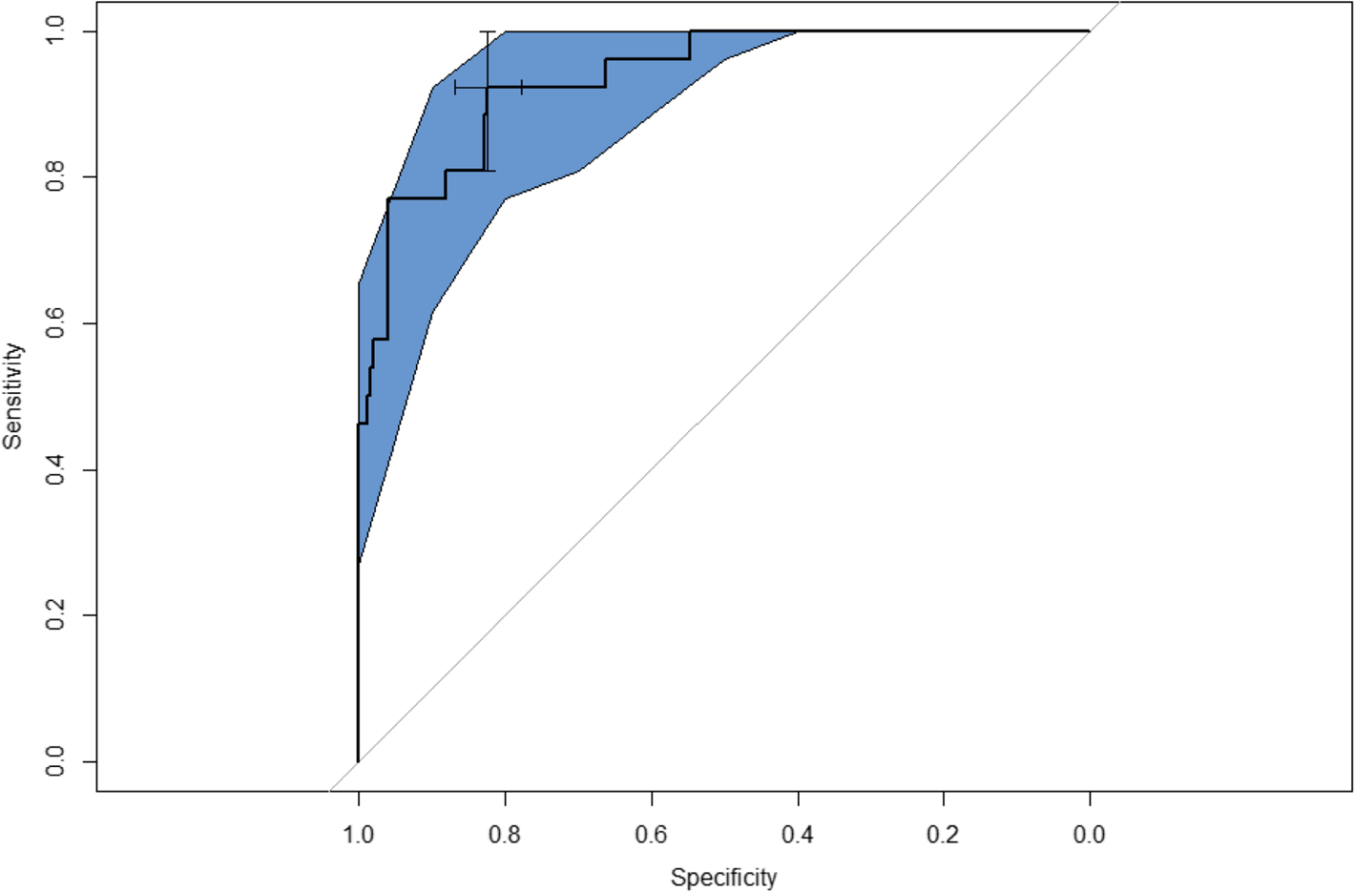
*ROC curve* for the Ci/CSF ratio (signal intensity ratio of the vestibular cistern to that of the signal intensity of the CSF) between obstructive vestibular schwannomas and IAC meningiomas. When Ci/CSF was above 0.70, the tumour was more likely a meningioma

**Fig. 5:**
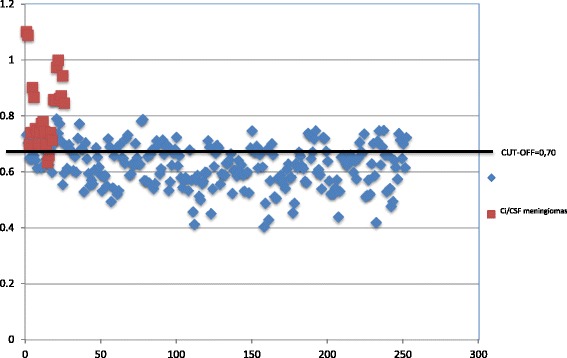
*Scattered dots graph* representing all the values of the Ci/CSF ratio in obstructive vestibular schwannomas (*blue dots*) and IAC meningiomas (*red dots*). The *black line* represents the optimal Ci/CSF ratio’s cutoff value of 0.70 to differentiate between the two groups with the highest sensitivity and specificity

**Fig. 6:**
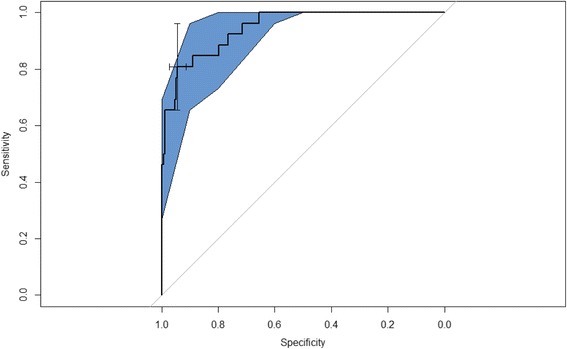
*ROC curve* for the Co/CSF signal intensity ratio (signal intensity ratio of the cochlea to that of the signal intensity of the CSF) between obstructive vestibular and IAC meningiomas. When Co/CSF was above 0.63, the tumour was more likely an IAC meningioma

IAC meningiomas presented with a decrease of the perilymphatic fluid signal with a Ci/CFS ratio of 0.81 ± 0.11 (mean ± SD) and a Co/CSF ratio of 0.76 ± 0.13, significantly different if compared with the control group (Pr(diff > 0) = 1, credibility interval [−0.22, −0.12] for the cistern and [−0.38 -0.34] for the cochlea). The signal decrease of the perilymphatic fluid was more moderate in meningiomas than in obstructive vestibular schwannomas and the Bayesian analysis was able to differentiate between those two tumours with Pr(diff > 0) = 1, [0.14 + 0.23]. The optimal cutoff point to differentiate between these tumours was Ci/CSF ratio of 0.70 in the vestibular cistern, with a ROC-AUC of 0.94 for the two observers combined (Figs. 4 and 5). For Ci/CSF ratio above 0.70, the tumour was more likely a meningioma, with a sensitivity of 92% and a specificity of 83%, a positive predictive value of 0.35 (24/68) and a negative predictive value of 0.99 (208/210). Regarding the cochlear signal, a cutoff point of Co/CSF ratio of 0.63 enabled the observers to properly differentiate between the two groups with a ROC-AUC of 0.9446 (Fig. 6). For a Co/CSF ratio above 0.63, the tumour was more likely a meningioma with a sensitivity of 81% and a specificity of 94%, a positive predictive value of 0.60 (21/35) and a negative predictive value of 0.98 (238/243).

**Fig. 7:**
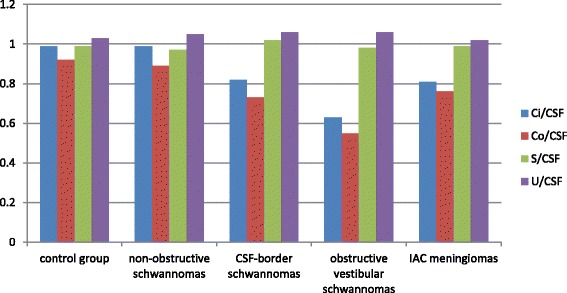
Comparison of perilymphatic signal intensity ratios (Ci/CSF and Co/CSF) and endolymphatic signal ratios (S/CSF for the saccular/CSF signal ratio and U/CSF for the utricular signal/CSF signal ratio) in the five studied groups. The mean Ci/CSF and Co/CSF ratios were the lowest in obstructive vestibular scwannomas. The mean ratios for the perilymphatic signal (Ci/CSF and Co/CSF) in IAC meningiomas were significantly lower than in the control group, but not as low as those observed in obstructive vestibular schwannomas. There were no significant signal changes in the endolymph (S/CSF and U/CSF) in any of the studied groups

Among the vestibular schwannomas that we subdivided in three groups, the perilymphatic signal decrease was correlated to the degree of IAC obstruction by the schwannoma (Fig. 7). Obstructive vestibular schwannomas (group 4) presented the lowest signal intensity of both the vestibular cistern and the cochlea in all 129 patients. Less-obstructive CSF-border schwannomas (group 2) had a more moderate decrease of the perilymphatic signal with a Ci/CSF ratio of 0.82 ± 0.11 in the cistern, a Co/CSF ratio of 0.73 ± 0.12 in the cochlea and a significant difference compared with groups 1 and 3 and the normal population (Pr(diff > 0) = 1). There was no significant difference between the perilymphatic signal decrease between CSF-border schwannomas and IAC meningiomas either for the Ci/CSF ratio in the vestibular cistern (Pr(diff > 0) = 0.49, [−0.05, 0.05]) or for Co/CSF ratio in the cochlea (Pr(diff > 0) = 0.81, credibility interval [−0.02, 0.07]). Non-obstructive schwannomas did not show any significant changes in the perilymphatic fluid in either the vestibular cistern or the cochlea compared to the control group with a Ci/CSF ratio of 0.09 ± 0.10 (Pr(diff > 0) = 0.55, [−0.03, 0.03]) and a Co/CSF ratio of 0.89 ± 0.08 (Pr(diff > 0) = 0.35, [−0.06, 0.02]).

There were no significant changes of the endolymphatic signal intensity among the five study groups (Fig. 7). There were no significant differences between the mean S/CSF ratio (0.97 ± 0.09 in non-obstructive schwannomas compared with 0.99 ± 0.10 in the control group; 1.01 ± 0.09 in CSF-border schwannomas; 0.98 ± 0.10 in obstructive vestibular schwannomas; 0.99 ± 0.10 in IAC meningiomas) or the mean U/CSF ratio (1.05 ± 0.08 in non-obstructive schwannomas compared with 1.03 ± 0.09 in the control group; 1.07 ± 0.10 in CSF border schwannomas; 1.06 ± 0.09 in obstructive vestibular schwannomas; 1.02 ± 0.09 in IAC meningiomas).

## Discussion

The aim of this study was to analyse the perilymphatic signal as a diagnostic tool to differentiate obstructive vestibular schwannomas from IAC meningiomas. In almost all cases, obstructive vestibular schwannomas presented with an important decrease of the perilymphatic signal in both anterior and posterior labyrinth. The signal decrease was more important than the one observed in almost all IAC meningiomas and was also significantly different when compared with the perilymphatic signal of healthy volunteers. When the Ci/CSF ratio was set above 0.70 (or with Co/CSF > 0.65) the tumour was most likely a IAC meningioma, with 92% sensitivity and 83% specificity.

A visual assessment of the perilymphatic fluid signal is useful and should be performed when reading an MRI for the diagnosis of a tumour of the cerebello-pontine angle. Obstructive vestibular schwannomas always presented with a perilymphatic signal rated as 2 (as low as the tumour signal on T2W images), while all IAC meningiomas had a perilymphatic signal visually rated either 0 or 1 (but never as low as the signal of the tumour on T2W images). The visual assessment should not lengthen the interpretation time. Ratio calculation, being slightly more time-consuming, could be reserved for cases in which the diagnosis remains uncertain.

The meningiomas are far less frequent that vestibular schwannomas in the cerebello-pontine angle (10–15% versus 85–90%) and those tumours only invade the IAC in 10–22% of cases. This explains the relatively small number of meningiomas included in our study. The IAC meningiomas and the obstructive vestibular schwannomas included in our study were comparable in volume and 11% of the patients underwent surgery, which confirmed the diagnosis.

The MRI features described here have a biological explanation, related to an increased protein concentration in the perilymph in the case of vestibular schwannomas as described by Silverstein et al. and Palva et al. [13, 17]. The FIESTA-C sequence is sensitive to changes in fluid protein concentration. As a consequence, an increase in the protein concentration in the perilymph, induces a decrease of the perilymphatic signal on a T2W gradient-echo sequence [13, 17]. This signal drop of the perilymph, due to increased protein concentrations, could have be seen with any other T2W gradient-echo SSFP sequence at 3T, such as the true fast imaging with steady state precession (trueFISP) or the constructive interference steady state (CISS) sequence. In 2013, in the only study regarding the use of a FIESTA sequence, Ishikawa et al. [18] described a drop in the vestibular cistern signal in 25 cases of vestibular schwannomas. Most of the research on the differentiation between vestibular schwannomas and IAC meningiomas, including those from Bhadelia et al. [19], Kim et al. [20] and Lee et al. [21], were based on an analysis of the labyrinth using FLAIR sequences, in which an elevation of perilymphatic protein concentrations leads to an increased signal [19–21]. In their studies, Kim et al. on one hand, and Bhadelia et al. on the other, only studied the cochlear signal. Ishikawa et al. [18] only analysed the vestibular signal. In our study, we analysed both structures, as did Lee et al. [20] who studied 34 vestibular schwannomas and described an increased labyrinthine signal on FLAIR images. In our compartmental analysis, not only did we find that an increased perilymphatic concentration may be observed in both cistern and cochlea at the same time, but we also found concordant perilymphatic signal ratios in both anterior and posterior labyrinth.

On histological sections, the membranous cochlear canal containing endolymph is very small, ten times smaller than the bony cochlear canal containing perilymph, and is not seen on MRI unless it is pathologically dilated [28]. Thus, we chose to consider the fluids of the cochlea as perilymph, like other authors did [18–21]. The lateral semi-circular canal acts as an anatomical landmark on MRI, and although it would have been easy to visually analyse it, any measurement would be challenging because of its small size. Therefore, we chose to analyse only structures for which both visual and quantitative analysis could easily be performed [18–21].

The more moderate decrease of the perilymphatic signal in IAC meningiomas also has a biological explanation, due to a more moderate increase of the protein concentrations in the perilymph of IAC meningiomas compared with vestibular schwannomas, as described by Palva et al. and O’Connor et al. [14, 16, 17]. O’Connor et al. found a significantly reduced protein concentration in the presence of IAC meningiomas (13 g/l) if compared with that in the presence of vestibular schwannomas (29.6 g/l) [14]. Thomsen and Bhadelia suggested that a failure of the perilymphatic drainage in IAC tumours was the cause of these abnormalities [15, 19]. This being so, the favoured physiopathological hypothesis for these changes is a blockage of the perilymph drainage, secondary to a compression of the perineurium and epineurium of the vestibulocochlear nerve sheath by the tumour. This compression occurs at earlier stages in vestibular schwannomas (which develops directly in the sheath) than in meningioma (which originate remotely from neural structures). The more moderate drop in perilymphatic signal in CSF-border vestibular schwannomas is then related to a lowered compression on neural structure by a less obstructive tumour, slightly distant from the IAC walls.

To our knowledge, no one has ever studied the endolymphatic signal in cerebello-pontine angle tumours. Silverstein reported an endolymphatic protein concentration of 180 mg/l, which is ten times lower than that in the perilymph [29]. Various studies demonstrated that the endolymph drains through the membranous endolymphatic canal in the vestibular aqueduct [30–34]. The absence of changes of the endolymphatic signal is therefore explained by its drainage, taking place outside the IAC, thus not influenced by the presence of a tumour in the IAC.

In conclusion, the proper differentiation between vestibular schwannoma and IAC meningioma is clinically relevant, especially for the choice of surgical approach. In obstructive vestibular schwannomas, the perilymph always appeared as hypointense as the tumour on a T2W gradient-echo SSFP sequence at 3T. This provides an additional tool for the differentiation between these two tumours, which may be useful to overcome the insufficiency of the morphological analysis itself, as the perilymphatic signal in meningiomas is either normal or just slightly lowered, but never as low as in vestibular schwannomas. An additional ratio calculation, with a cutoff of 0.70 for Ci/CSF ratio, enables the reader to differentiate between these two tumours. The reading of MRI images in tumours of the internal auditory canal and cerebello-pontine angle should include quick visual analysis of the inner ear fluids on a T2W gradient-echo sequence at 3T, as it does not lengthen the interpretation time, but strengthens the diagnosis.

## Abbreviations

AUC: Area under the curve
Ci/CSF: ratio between the signal intensity of the cistern on the signal intensity of the CSF
Co/CSF: ratio between the signal intensity of the cochlea on the signal intensity of the CSF
CSF: cerebrospinal fluid
FIESTA: fast imaging employing steady state acquisition
FLAIR: fluid attenuation inversion Recovery
FOV: field of view
IAC: internal auditory canal
ROC: receiver operating characteristic
ROI: region of interest
S/CSF: ratio between the signal intensity of the saccule and the signal intensity of the CSF
TE: echo time
TR: repetition time
TrueFISP: true fast imaging with steady state precession
U/CSF: ratio between the signal intensity of the utricle and the signal intensity of the CSF.
